# Unravelling the genes forming the wing pattern supergene in the polymorphic butterfly *Heliconius numata*

**DOI:** 10.1186/s13227-019-0129-2

**Published:** 2019-08-08

**Authors:** Suzanne V. Saenko, Mathieu Chouteau, Florence Piron-Prunier, Corinne Blugeon, Mathieu Joron, Violaine Llaurens

**Affiliations:** 1 Institut de Systématique, Evolution et Biodiversité, UMR 7205 (CNRS, MNHN, Sorbonne Université, EPHE), Muséum National d’Histoire Naturelle CP50, 57 rue Cuvier, 75005 Paris, France; 2 Laboratoire Ecologie, Evolution, Interactions Des Systèmes Amazoniens (LEEISA), USR 3456, CNRS Guyane, Université De Guyane, 275 route de Montabo, 97334 Cayenne, French Guiana; 3 Genomic Facility, Institut de Biologie de l’Ecole normale superieure (IBENS), École normale supérieure, CNRS, INSERM, PSL Université Paris, 75005 Paris, France; 4 Centre d’Ecologie Fonctionnelle et Evolutive, UMR 5175 CNRS-Université de Montpellier, École Pratique des Hautes Études, Université Paul Valéry, 34293 Montpellier 5, France

**Keywords:** Supergene, Wing pattern, Lepidoptera, *Heliconius* butterflies, Transcriptome, RNA sequencing, Gene expression

## Abstract

**Background:**

Unravelling the genetic basis of polymorphic characters is central to our understanding of the origins and diversification of living organisms. Recently, supergenes have been implicated in a wide range of complex polymorphisms, from adaptive colouration in butterflies and fish to reproductive strategies in birds and plants. The concept of a supergene is now a hot topic in biology, and identification of its functional elements is needed to shed light on the evolution of highly divergent adaptive traits. Here, we apply different gene expression analyses to study the supergene *P* that controls polymorphism of mimetic wing colour patterns in the neotropical butterfly *Heliconius numata*.

**Results:**

We performed de novo transcriptome assembly and differential expression analyses using high-throughput Illumina RNA sequencing on developing wing discs of different *H. numata* morphs. Within the *P* interval, 30 and 17 of the 191 transcripts were expressed differentially in prepupae and day-1 pupae, respectively. Among these is the gene *cortex*, known to play a role in wing pattern formation in *Heliconius* and other Lepidoptera. Our in situ hybridization experiments confirmed the relationship between *cortex* expression and adult wing patterns.

**Conclusions:**

This study found the majority of genes in the *P* interval to be expressed in the developing wing discs during the critical stages of colour pattern formation, and detect drastic changes in expression patterns in multiple genes associated with structural variants. The patterns of expression of *cortex* only partially recapitulate the variation in adult phenotype, suggesting that the remaining phenotypic variation could be controlled by other genes within the *P* interval. Although functional studies on *cortex* are now needed to determine its exact developmental role, our results are in accordance with the classical supergene hypothesis, whereby several genes inherited together due to tight linkage control a major developmental switch.

**Electronic supplementary material:**

The online version of this article (10.1186/s13227-019-0129-2) contains supplementary material, which is available to authorized users.

## Background

The evolution of complex phenotypes requiring the coordinated diversification of several traits is a puzzle. The persistence of intraspecific polymorphisms with several differentiated variants segregating within a single interbreeding population facilitates the identification of genes and epistatic interactions that control the developmental switches involved in their emergence. Unravelling the genetic architecture of polymorphic traits is therefore central to our understanding of the origins and evolutionary diversification of complex phenotypes.

The maintenance of discrete adaptive morphs that require the co-variation of multiple phenotypic modalities is surprising because interbreeding among morphs should lead to recombination between co-adapted loci, shuffling allelic combinations, and resulting in maladaptive intermediates. Recently, the dissection of several cases of balanced polymorphism has revealed a genetic architecture where a single Mendelian locus coordinates major phenotypic changes (see [1] for a review). These polymorphic loci could either be composed of a single causative gene with diverse pleiotropic effects, or of several tightly linked genes acting on one modality each, i.e. so-called “supergenes”. Supergenes are usually described as clusters of two or more loci, each affecting a different morphological or behavioural trait; tight physical linkage and/or chromosomal inversions suppress recombination, such that multiple characters are inherited as a single Mendelian locus [2, 3]. This architecture prevents suboptimal allelic combinations and results in the long-term co-existence of multiple well-differentiated variants (e.g. [4]), or conversely in reproductive isolation and speciation among ecotypes (e.g. [5]). In over 80 years since the proposal of this idea [6], supergenes have been reported in a wide range of complex polymorphisms, from adaptive colouration in butterflies and fish, through social structure in ants, to reproductive and behavioural strategies in animals and plants (e.g. [7–12]). The emergence of such genetic architecture, however, remains puzzling.

Despite the panoply of complex polymorphisms associated with supergenes, molecular evidence for the involvement of multiple genetic elements remains scarce. The individual genetic components have been characterised in detail only for the self-incompatibility supergenes in flowering plants [13, 14]. For the polymorphism in wing colour pattern in *Papilio polytes* butterflies, controlled by a large inversion, supergene architecture was presumed for long time. However, recent gene expression data showed that this variation is controlled mostly by a single transcription factor *doublesex*, located within the inverted region and acting like a major developmental switch between different morphs via alternative splicing [15]. It remains unclear whether multiple genetic elements are functionally involved in this wing colour polymorphism. The concept of supergene is now a hot topic in biology, and identification of its functional elements (distinct genes vs. mutations in a single gene) is needed to shed light on the evolution of highly divergent polymorphic traits. Here, we apply different gene expression analyses to study the supergene that controls a well-documented polymorphism in a classical ecological model, the wing colour variation observed within populations of the neotropical butterfly *H. numata*.

*Heliconius* are chemically defended butterflies famous for their colourful wing patterns acting as warning signal towards predators [16]. Most species are involved in Müllerian mimicry associations with other locally abundant unpalatable species. For example, *H. numata* exhibits extraordinary resemblance to a number of species from the distantly related genus *Melinaea*, which diverged from the genus *Heliconius* over 90 million years ago [17]. A handful of genetic loci of major effect explain most of the variation in wing pattern within and among the majority of *Heliconius* species (reviewed in [18]). Recent studies have revealed the molecular identity the major loci: the transcription factor *optix* is responsible for turning on and off red, orange, and brown pattern elements [19], while the presumed cell cycle regulator *cortex* acts as a switch for black [20], and the regulatory gene *aristaless* controls white/yellow colours [21]. The morphogen *WntA*, in turn, controls the size and shape of the elements switched on and off by the first two loci [22]. These and a few other, yet unidentified loci belong to the genetic tool-kit that controls variation and produces both convergent and divergent wing colour patterns in *Heliconius* and other butterflies [23, 24].

**Fig. 1:**
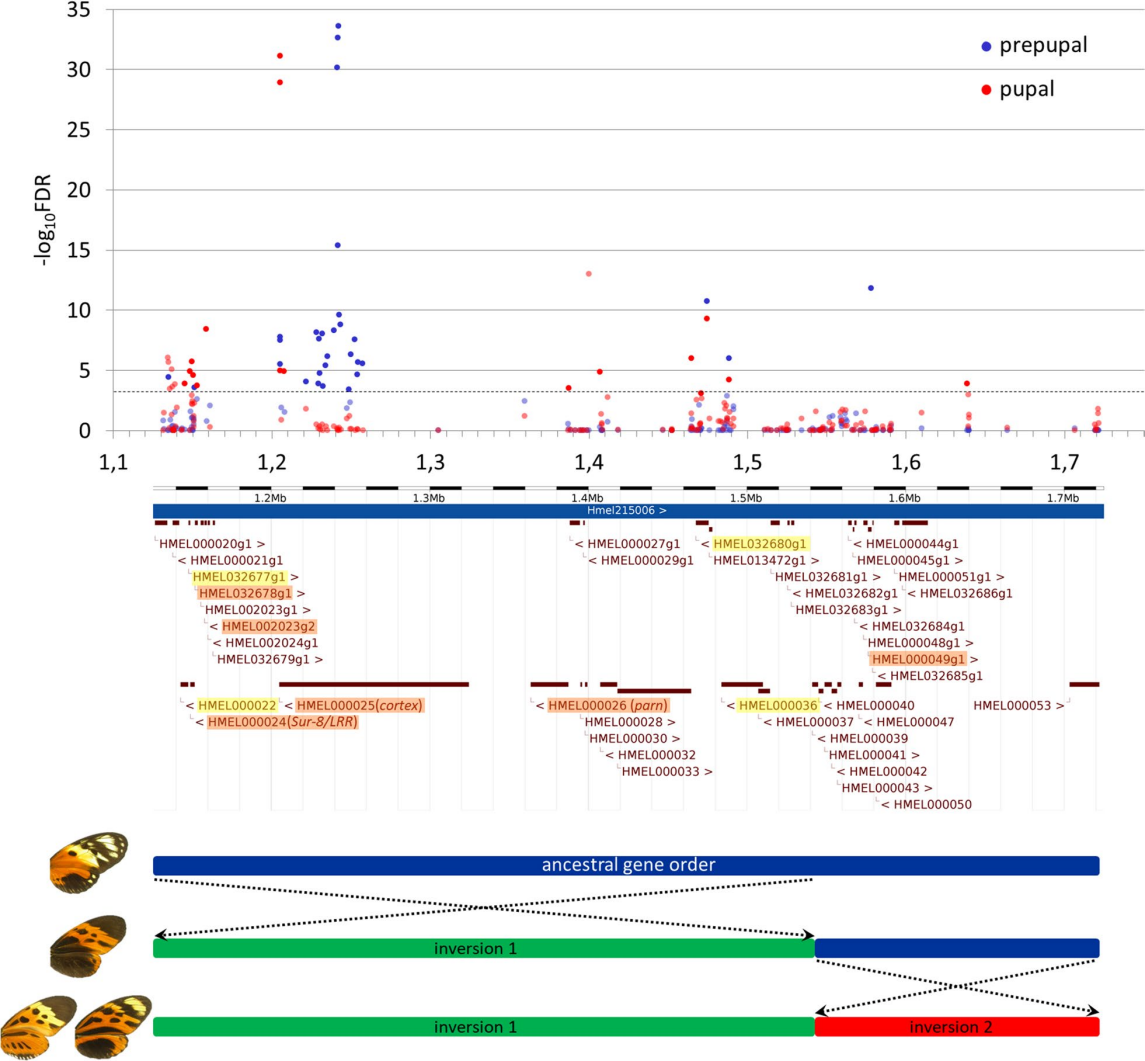
Differential gene expression across the *P* supergene region. Expression differences in prepupal (blue) and day-1 pupal (red) wing discs for 191 transcripts with a BLASTn hit to the interval between 1,126,790 bp (gene *Hmel000020*) and 1,722,158 bp (gene *Hmel000053*) on *H. melpomene* genomic scaffold 215006 (*cf.* [35]). Significantly (FDR-adjusted *p* value < 0.001, see grey dotted threshold line) differentially expressed transcripts are indicated with bright colours. The corresponding *H. melpomene* genes are highlighted with orange (in case the differentially expressed transcripts match exons and introns) or yellow (in case the transcripts match introns only). The four examined morphs belong to three different gene orders: (1) ancestral (*silvana*), (2) first inversion (*bicoloratus*), and (3) first + second inversions (*tarapotensis* and *aurora*)

In contrast to most *Heliconius* species where all individuals display the same colour pattern within each locality, *H. numata* shows a stable local polymorphism of wing colour pattern, with up to seven distinct morphs co-occurring in a single population [25]. This polymorphism is almost entirely determined by a single locus, the supergene *P*, found on linkage group 15 and characterised by long-range haplotypes in complete linkage disequilibrium [26]. Recombination in this region is suppressed due to the presence of two chromosomal inversions arranged in three distinct gene orders co-existing within populations [7] (Fig. 1). For example, the morph *silvana* is controlled by haplotype *P*^*sil*^ which corresponds to the standard, ancestral gene arrangement, shared with most *Heliconius* species, while *bicoloratus* (*P*^*bic*^) is determined by the first inversion, a 400-kb segment containing genes from *Hnum000020* to *Hnum000040*; the second adjacent inversion, a 180-kb segment containing genes from *Hnum000041* to *Hnum000053*, forms yet another gene order associated with several other morphs such as *tarapotensis* (*P*^*tar*^) and *aurora* (*P*^*aur*^). This supergene architecture presumably locks together the genetic combinations producing mimetic colour patterns: haplotypes controlling distinct morphs rarely recombine, therefore maintaining co-adapted alleles together. Remarkably, the *P* locus contains the gene *cortex*, the expression of which in final instar larval wing discs is associated with some black elements on the hindwings of *H. numata* and *H. melpomene* [19]. Is *cortex* the unique factor controlling the developmental switch between mimetic patterns in *H. numata*, similar to *doublesex* in *P. polytes*? Or are other genes within the *P* locus involved in pattern variation as well, making the *P* a classical supergene, i.e. “co-adapted combination of several genes locked in inverted section of chromosome” [27]? Here, we apply different gene expression analyses to investigate the role of *cortex* and other genes within the *P* supergene in controlling the variation of *H. numata* wing patterns. First, we perform RNA sequencing and differential expression analysis of the supergene *P* genes at relevant stages of colour pattern development. We then investigate the differences in spatial expression patterns of candidate genes in the developing wing discs.

## Methods

### Butterfly rearing

*Heliconius numata* butterflies were collected in Tarapoto, Peru. Adults were fed with sugar water and pollen, and provided with *Passiflora caerulea* for egg deposition. Larvae were reared on *P. caerulea* and *P. edulis* plants.

### Genotyping assays

The genotype of each individual at the supergene *P* was determined using PCR-based genotyping assays based on intron length and SNP polymorphisms at the *cortex* gene (modified protocol of Chouteau et al. [28]). Genomic DNA was extracted from the bodies using Qiagen DNeasy Blood & Tissue kit *cf*. the manufacturer’s instructions. PCRs with forward 5′-CGTAGCGACCCGAGATTCTT and reverse 5′-ATACATGGCCACAGTTGATTC primers were carried out with 5 min at 94 °C, followed by 35 cycles of 25 s at 94 °C, 25 s at 58 °C, and 60 s at 72 °C, and a final elongation phase at 72 °C for 5 min. Gel electrophoresis was used to determine the size of the resulting PCR products: 374 bp (present only in *P*^*sil*^ haplotype), 925 bp (present only in *P*^*bic*^), or 644 bp (present in *P*^*bic*^, *P*^*tar*^, and *P*^*aur*^). The PCR products were also sequenced directly with the same primers to determine the genotype on the basis of diagnostic SNPs.

### Library preparation and RNA sequencing

Individuals for RNA sequencing were collected from second generation of crosses between butterflies displaying *aurora*, *bicoloratus*, *silvana*, and *tarapotensis* phenotypes (Fig. 1). Wing discs were dissected in PBS from prepupae and day-1 pupae and stored in RNAlater. We used wing disc from prepupae with genotypes *P*^*aur*^*/P*^*aur*^ (*n* = 4), *P*^*bic*^*/P*^*bic*^ (*n* = 4), *P*^*bic*^*/P*^*tar*^ (*n* = 3), and *P*^*tar*^*/P*^*tar*^ (*n* = 3), and from day-1 pupae with genotypes *P*^*aur*^*/P*^*aur*^ (*n* = 4), *P*^*sil*^*/P*^*sil*^ (*n* = 3), *P*^*tar*^*/P*^*tar*^ (*n* = 3), resulting in 24 samples in total.

One forewing and one hindwing of each individual were homogenised in 350 µl of RTL buffer with the Tissue Lyser (Qiagen, Hilden, Germany). Total RNA was extracted according to the manufacturer’s protocol (RNeasy Mini kit, Qiagen, Hilden, Germany) and eluted in 30 µl of RNase-free water. To avoid genomic contamination, RNase-free DNase treatment (Qiagen, Hilden, Germany) was performed during RNA extraction. RNA quality and concentration were measured with a Qubit 2.0 Fluorometer (Life Technologies) and a 2100 Bioanalyzer (Agilent Technologies).

Library preparation and Illumina sequencing were performed at the Ecole Normale Supérieure core genomic facility (Paris, France). Messenger (polyA+) RNAs were purified from 1 µg of total RNA using oligo(dT). Libraries were prepared from pools of fore- and hindwing RNA of each individual using the TruSeq Stranded mRNA kit (Illumina). Twenty-four libraries (multiplexed by six on four flowcell lanes) were sequenced on Illumina HiSeq 1500 sequencer using 51 cycles per run, yielding 42 ± 15 million single 50-bp passing Illumina quality filter reads per sample. Sequencing lanes were randomized among samples.

### de novo transcriptome assembly and gene expression analyses

All bioinformatics analyses were performed on the Galaxy server [29] of BioInformatics Platform for Agro-ecosystems Arthropods (BIPAA) of French National Institute for Agricultural Research (INRA) in Rennes, France. Low-quality (< Q30) reads, adaptor sequences, and ribosomal RNA-like sequences were removed with Prinseq [30], Cutadapt [31], and riboPicker [32] tools, respectively. A total of ~ 959,842,465 high-quality clean reads from all samples were used to de novo assemble the wing disc transcriptome with the software Trinity [33] and following parameters: SS_lib_type = F, kmer_size = 25, max_pct_stdev = 100, minimum contig length = 200 bp. Quality of the assembly was evaluated by estimating transcript abundance using the RSEM method [34] and subsequently computing N50 and ExN50 statistics. The latter takes into account the expression levels of each contig and is therefore a more suitable contig length metric for transcriptomes. To identify transcripts with orthologs in *H. melpomene*, all RNA sequences were aligned to the Hmel2_cds and Hmel2_scaffolds databases (downloaded from Lepbase v4 [35]) using NCBI BLASTn.

Reads from each sample were mapped to the assembled transcriptome using Bowtie2 [36]. Mapped reads for each transcript were counted with samtools idxstats [37]. Read counts were used to identify differentially expressed transcripts with the Bioconductor EdgeR package, as it performs best for datasets with small numbers of replicates [38].

Comparison of differential expression in genes located within the supergene with respect to the genes located in the rest of the genome were performed using gene set permutations test using the gene set enrichment analysis described in Subramanian et al. [39]. For each pair of phenotype within the two developmental stages, we used the 191 transcripts mapped to the supergene *P* has a gene set and performed 1000 permutations to test whether the differential expression ranking significantly differed to the rest of the genome, using the GSEA software (http://software.broadinstitute.org/gsea/index.jsp).

### Wing disc in situ hybridizations (ISH)

Fragments (~ 300 to 700 bp long) of 28 candidate genes expressed within the *P* interval (see Additional file 1: Table S1) were cloned into pCRII dual-promoter vector using the TA cloning kit (Life Technologies). Plasmids were isolated with QIAprep Spin Miniprep Kit (Qiagen) and used as template for PCR reactions with vector primers M13F and M13R. The amplified products were cleaned with QIAquick PCR purification kit (Qiagen) and used for SP6 or T7 transcription. Digoxigenin-labelled riboprobes were synthesised using SP6 and T7 RNA polymerases and DIG RNA labelling mix (Roche Applied Science).

Individuals used for ISH came from a *H. numata* stock (a mix of different morphs) maintained in the greenhouse at MNHN in Paris. At least five individuals of each (*silvana*, *tarapotensis*, and *bicoloratus*) morph were used for ISH. Wing discs were dissected in PBS from last instar larvae, fixed in 4% formaldehyde in PBS, gradually dehydrated and stored in methanol. ISH was performed *cf*. the protocol described previously in Martin and Reed [40]. Briefly, wing discs were gradually rehydrated, incubated 5 min with 25 μg/ml proteinase K, post-fixed with 5.5% formaldehyde in PBS, and incubated in a standard hybridization buffer supplemented with 1 g/l glycine and 30 ng/ml riboprobe for 20–24 h at 63 °C. For secondary detection of the probe, wing discs were incubated in a 1:3000 dilution of anti-digoxigenin alkaline phosphatase Fab fragments and stained with BM Purple (Roche Applied Science) for 3–6 h at room temperature. Stained wing discs were photographed with a Leica DFC420 digital camera mounted on a Leica Z6 APO stereomicroscope.

## Results

### Assembly of the *H. numata* wing disc transcriptome

We performed a de novo transcriptome assembly using Illumina RNA sequencing data on fore- and hindwings of 24 individuals (14 prepupae and 10 day-1 pupae). Trinity yielded 53,719 transcripts with a cumulative length of 34.37 Mb and the mean, median, and maximal transcript length of 640 bp, 367 bp, and 33,692 bp, respectively. A large part of the assembled transcripts (20,406 out of 53,719) fall within the size range of 201–300 bp; 9162 transcripts are longer than 1000 bp, with 12 exceeding 10 kb. We calculated the transcript N50 statistics of 980 bp. However, when excluding the transcripts expressed at very low levels, the maximal N50 value of 1356 bp was found for Ex = 88, i.e. 50% of the total assembled nucleotides are included in the top 88% abundant transcripts (*N* = 7331) after normalising for read coverage. About 92.5% of the assembled sequences represent unique transcripts, whereas 4004 represent different isoforms of 2957 genes, some of which have up to 20 splicing variants. The transcriptome is available for download at Transcriptome Shotgun Assembly Sequence Database of NCBI (NCBI BioProject PRJNA555830).

A high-quality genome assembly is currently unavailable for *H. numata*. The assembly consists of more than 20,000 scaffolds with N50 of 61.3 kb (*cf.* Lepbase v4 [35]). Therefore, we performed the analyses using the reference genome assembly Hmel2 (795 scaffolds, N50 = 2.1 Mb [41]) of the closely related *H. melpomene*, diverged from *H. numata* around 4 Mya [42]. Of the 53,719 transcripts present in the *H. numata* wing disc transcriptome, more than 99% (53,301) could be aligned to the *H. melpomene* genomic scaffolds. However, only 27,999 *H. numata* transcripts were identified as orthologous to 12,835 out of 21,661 transcripts in *H. melpomene* coding DNA sequence (CDS) database. Such discrepancy might come from important alternative splicing variations between species, or incompleteness of *H. melpomene* CDS database.

We identified 191 transcripts corresponding to 169 unigenes (i.e. 157 transcripts with just one isoform and 12 transcripts with 2–8 isoforms) in the *H. numata* transcriptome that were aligned to the interval between 1,126,790 bp (gene *Hmel000020*) and 1,722,158 bp (gene *Hmel000053*) on *H. melpomene* genomic scaffold 215006, i.e. the interval homologous to the *P* supergene. Of these 191 transcripts, 72 have a BLASTn hit to one of the 36 (out of the 41 present in this interval) predicted genes in the *H. melpomene*, with multiple short transcripts being part of the same gene. Hence, the majority of the genes in the *P* supergene are expressed in the wing discs of *H. numata* at the prepupa and/or day-1 pupa stages, when wing pattern formation occurs. The genes not found in the transcriptome, and therefore not expressed at the relevant stages, are *Hnum000051*, *Hnum002023g1*, *Hnum032679*, *Hnum032683*, and *Hnum032685*. None of them is annotated for gene ontology terms.

### Differential expression within the *P* supergene

To identify the genes within the *P* locus potentially associated with wing pattern development, we performed differential expression analysis using wing disc samples of different morphs (3–4 biological replicates per morph) at two developmental stages (prepupa and day-1 pupa), known to be important in wing pattern formation in other butterflies [43]. Principal component analysis showed a clear segregation of prepupal and day-1 pupa samples (circles vs. squares in Additional file 1: Figure S1), with a single *tarapotensis* pupa as an outlier. Overall, 1048 (in prepupa) and 3488 (in day-1 pupa) of the 53,719 transcripts were identified as differentially expressed between morphs using a false discovery rate (FDR) adjusted *p* value of < 0.001 and at least a twofold change in the expression level. Neither *WntA* nor *optix*, two major wing patterning genes in Lepidoptera (acting at larval and late pupal stages, respectively [19, 22]), and unlinked to the *P* interval, show differences in expression levels among *H. numata* morphs. The differential expression within the supergene was generally more marked than in the rest of the transcriptome, as revealed by gene set enrichment analysis (see Additional file 1: Table S2). Within the *P* interval, 30 and 24 of the transcripts mapping to the supergene were indeed differentially expressed among morphs at the prepupa and day-1 pupa stages, respectively (bright blue and red dots in Fig. 1).

However, seven of the 24 transcripts had very high expression levels in a single *tarapotensis* day-1 pupa (an outlier in the PCA plot, see Additional file 1: Figure S1) in comparison with two other samples of the same morph and therefore were not taken into consideration in further analyses (pale red dots above the threshold line in Fig. 1). Moreover, the majority of differentially expressed transcripts within the *P* interval generally have low expression levels as indicated by negative logCPM values (Table 1).

**Table 1:** List of contigs in the *P* region expressed differentially among *H. numata* morphs at prepupa and/or day-1 pupa stages

Contig	Median position in scaf215006	Best BLASTn hit in Hmel genome (version Hmel2) scaf215006	Prepupa (logFC vs. aur)			Log CPM	Day-1 pupa (logFC vs. aur)		Log CPM	DE significant (FDR < 0.001)
			bic	bic/tar	tar		sil	tar		
comp141924_c0_seq1	1,135,136	Intergenic	− 3.0	− 2.8	− 3.4	− 1.82	0.5	1.4	− 1.36	Prepupa
comp96881_c0_seq1	1145125	HMEL000022 intron 2	0.9	− 1.0	− 0.9	− 4.61	− 2.9	− 2.4	− 1.09	Day-1 pupa
comp94831_c0_seq1*	1,148,671	HMEL032677 intron 3	3.4	3.2	2.2	− 1.97	− 3.0	0.9	− 0.18	Day-1 pupa
comp90289_c0_seq1*	1,149,824	HMEL000024 intron 2	3.4	2.9	1.7	− 2.73	− 5.1	− 0.5	− 0.47	Day-1 pupa
comp35900_c0_seq2*	1,150,543	HMEL000024 CDS	0.9	0.6	1.0	− 0.80	− 2.6	1.0	− 0.17	Day-1 pupa
comp35224_c0_seq1	1,151,211	HMEL000024 CDS	2.1	2.5	2.9	1.59	− 1.1	0.6	2.67	Prepupa
comp284389_c0_seq1*	1,153,003	HMEL032678 CDS	− 2.1	1.7	3.1	− 2.36	2.9	− 1.4	− 2.74	Day-1 pupa
comp292494_c0_seq1	1,158,885	HMEL002023g2 CDS	− 1.7	− 2.6	− 5.8	− 5.21	− 4.6	− 9.5	− 1.67	Day-1 pupa
comp45148_c0_seq1	1,205,329	Cortex CDS	1.1	0.5	− 3.0	2.29	3.8	− 1.2	3.95	Both stages
comp45148_c0_seq2	1,205,329	Cortex CDS	0.9	0.2	− 3.3	2.21	3.9	− 1.4	4.00	Both stages
comp217022_c0_seq1	1,205,576	Cortex intron 8	4.6	4.1	0.0	− 2.05	5.1	− 0.4	− 3.48	Both stages
comp388498_c0_seq1	1,207,870	Cortex intron 1	− 1.9	0.5	− 6.0	− 4.13	3.7	− 0.6	− 2.59	Day-1 pupa
comp407410_c0_seq1	1,221,611	Cortex intron 1	3.5	3.6	− 4.1	− 2.91	3.9	− 2.4	− 5.73	Prepupa
comp398064_c0_seq1	1,228,279	Cortex intron 1	8.5	8.1	0.0	− 2.22	2.4	1.2	− 4.48	Prepupa
comp601770_c0_seq1	1,229,498	Cortex intron 1	4.4	3.2	− 3.2	− 3.43	3.4	0.0	− 8.88	Prepupa
comp415437_c0_seq1	1,230,060	Cortex intron 1	5.2	4.0	− 0.8	− 1.78	2.5	2.2	− 5.05	Prepupa
comp423466_c0_seq1	1,230,537	Cortex intron 1	7.4	6.9	0.0	− 3.40	− 3.2	0.5	− 6.95	Prepupa
comp407210_c0_seq1	1,231,923	Cortex intron 1	8.7	7.3	0.0	− 2.35	− 3.7	1.6	− 5.67	Prepupa
comp467980_c0_seq1	1,232,299	Cortex intron 1	3.8	3.0	− 0.5	− 2.38	0.1	1.0	− 4.12	Prepupa
comp472259_c0_seq1	1,234,155	Cortex intron 1	4.1	2.7	− 4.1	− 2.78	0.0	3.7	− 7.93	Prepupa
comp700774_c0_seq1	1,235,209	Cortex intron 1	8.0	6.3	0.0	− 3.16	0.1	1.1	− 6.32	Prepupa
comp321273_c0_seq1	1,239,339	Cortex intron 1	5.0	4.2	− 4.1	− 1.77	− 3.8	− 3.8	− 7.32	Prepupa
comp62384_c0_seq1	1,241,229	Cortex intron 1	8.9	7.6	− 3.2	− 4.18	− 3.2	− 3.2	− 7.93	Prepupa
comp31128_c1_seq1	1,241,616	Cortex intron 1	6.2	5.1	0.6	0.25	− 1.1	0.5	− 3.61	Prepupa
comp31128_c1_seq2	1,241,750	Cortex intron 1	9.0	7.9	− 3.2	1.20	− 0.8	− 1.0	− 5.15	Prepupa
comp83470_c0_seq1	1,242,240	Cortex intron 1	6.9	5.7	− 0.7	2.22	1.1	1.0	− 4.80	Prepupa
comp90384_c0_seq1	1,242,543	Cortex intron 1	5.5	4.0	− 4.2	− 1.42	− 2.4	1.9	− 6.95	Prepupa
comp382243_c0_seq1	1,243,573	Cortex intron 1	8.9	7.7	0.0	− 2.08	− 3.7	− 0.9	− 6.98	Prepupa
comp144920_c0_seq1	1,248,924	Cortex intron 1	− 0.1	− 1.3	− 8.3	− 2.05	− 6.6	− 1.8	− 4.34	Prepupa
comp615571_c0_seq1	1,250,088	Cortex intron 1	− 7.5	− 7.5	− 7.5	− 4.05	− 1.6	− 2.1	− 5.80	Prepupa
comp386445_c0_seq1	1,252,499	Cortex intron 1	3.5	1.5	− 5.8	− 1.80	− 0.4	0.1	− 6.33	Prepupa
comp527091_c0_seq1	1,254,035	Cortex intron 1	4.9	3.8	− 3.1	− 2.91	− 3.3	1.0	− 6.62	Prepupa
comp572350_c0_seq1	1,254,350	Cortex intron 1	4.3	3.0	− 4.1	− 2.69	− 3.7	− 0.9	− 6.98	Prepupa
comp331233_c0_seq1	1,257,539	Cortex intron 1	8.2	6.2	3.2	− 2.98	− 3.2	− 0.4	− 7.38	Prepupa
comp115525_c0_seq1*	1,387,318	HMEL000026 CDS	3.2	4.1	0.0	− 6.18	− 4.1	1.5	− 2.08	Day-1 pupa
comp8094_c0_seq1*	1,407,093	Intergenic	2.2	2.4	1.4	− 3.12	− 3.7	1.7	− 1.46	Day-1 pupa
comp54294_c0_seq1*	1,464,919	Intergenic	− 0.4	− 0.8	− 2.0	0.14	− 3.0	0.2	1.02	Day-1 pupa
comp131953_c0_seq1*	1,470,921	HMEL032680 intron 3	0.2	− 0.3	− 1.6	− 2.11	− 3.6	0.1	− 1.21	Day-1 pupa
comp223361_c0_seq1	1,474,761	HMEL032680 intron 1	− 8.6	− 8.6	− 8.6	− 2.96	− 9.1	− 9.1	− 2.11	Both stages
comp211996_c0_seq1	1,488,546	HMEL000036 intron 4	− 3.8	− 7.9	− 7.9	− 3.57	− 4.1	− 3.5	− 2.54	Both stages
comp166797_c0_seq1	1,578,231	HMEL000049 CDS	5.9	5.0	− 0.9	− 0.93	1.0	0.5	− 3.57	Prepupa
comp17881_c0_seq1	1,638,684	Intergenic	0.1	0.6	0.1	− 4.21	− 3.6	− 2.3	− 0.96	Day-1 pupa

Values represent − log_10_ fold changes (FC) of up- (positive) or downregulation (negative) in comparison with expression values in *aurora* morph (set to 0). LogCPM (i.e. log_2_ of counts-per-million) values are proportional to overall expression levels of each gene.*Genes located near the breakpoints of the first inversion

Five transcripts were expressed differentially at both developmental stages (Table 1). Three of them corresponded to the gene *cortex*, known to play a role in wing pattern formation in *Heliconius* and other Lepidoptera [20, 44], whereas the other two matched the introns of *Hmel032680* (predicted WD repeat-containing protein) and *Hmel000036* (predicted WAS protein homologue 1). Moreover, 22 transcripts expressed differentially in prepupae corresponded to the very large (more than 100 kb) first intron of *cortex*. At this stage, expression of *cortex* CDS and the majority of intron transcripts was highest in the *bicoloratus* homozygotes (*P*^*bic*^*/P*^*bic*^), intermediate in *bicoloratus* heterozygotes (*P*^*bic*^*/P*^*tar*^) and *aurora*, and lowest in *tarapotensis*. In day-1 pupae, expression of *cortex* CDS was highest in *silvana*, intermediate in *aurora*, and lowest in *tarapotensis*. However, only one transcript that corresponded to the first intron of *cortex* was differentially expressed at this stage. To establish whether the transcripts in the first intron of *cortex* represent alternative splicing variants (as has been observed in *H. melpomene* [20]), we performed BLASTn to *H. numata* scaffolds on Lepbase v4 [35] and examined 20-bp sequences flanking those transcripts for presence of GT–AG, indicative of intron splicing sites (see Additional file 1: Table S3). At least nine transcripts have both AG and GT in the flanking 5′ and 3′ regions, respectively, suggesting that those could be alternative splicing forms of *cortex*, whereas other transcripts could be distinct non-coding RNAs.

Furthermore, two transcripts corresponding to different parts of *Hnum000024* (predicted similar to Sur-8, a positive regulator of Ras signalling) CDS were expressed differentially in both prepupae (comp35224_c0_seq1, high in homozygote *bicoloratus* and *tarapotensis*, low in *aurora*) and day-1 pupae (comp35900_c0_seq2, high in *tarapotensis*, lowest in *silvana*), see Table 1. We also found *Hnum000049* (predicted protein coding) to be upregulated in *bicoloratus* homo- and heterozygotes at the prepupa stage. Gene *Hnum000026* (putative poly(A)-specific ribonuclease) was upregulated in *tarapotensis* and downregulated in *silvana* (in comparison with *aurora*) in day-1 pupae, whereas *Hnum032678* (predicted protein coding) showed the opposite pattern. Finally, gene *Hnum002023g2* (predicted protein coding) was downregulated in both *silvana* and *tarapotensis* at that stage.

### Differential expression in relation to supergene structure

Our experimental design allows for comparison of gene expression across different gene arrangements within the *P* supergene (Fig. 1). For instance, day-1 pupa stage allows for comparison of the ancestral gene arrangement in *silvana* versus both first (400-kb segment containing genes *Hnum000020*–*Hnum000040*) and second (180-kb segment containing genes *Hnum000041*–*Hnum000053*) inversions characteristic of *tarapotensis* and *aurora*. We found eight transcripts (indicated with * in Table 1) to be consistently up- or downregulated in *silvana* and showing the opposite pattern of expression in *tarapotensis* and *aurora*. Remarkably, the first four of those are located near the left breakpoint, whereas the other four are closer to the right breakpoint of the first inversion. Some of these transcripts correspond to introns of predicted genes or intergenic sequences. Hence, the first inversion does not only affect expression levels of the existing genes, but also introduces novel expression patterns. Morphs analysed at the prepupa stage, on the other hand, differ in the presence (in *tarapotensis* and *aurora*) or absence (in *bicoloratus*) of the second inversion. The presence of this genomic rearrangement is associated with decreased expression of transcript comp166797_c0_seq1 (*Hnum000049*).

### Variation in *cortex* expression patterns in larval wing discs

**Fig. 2:**
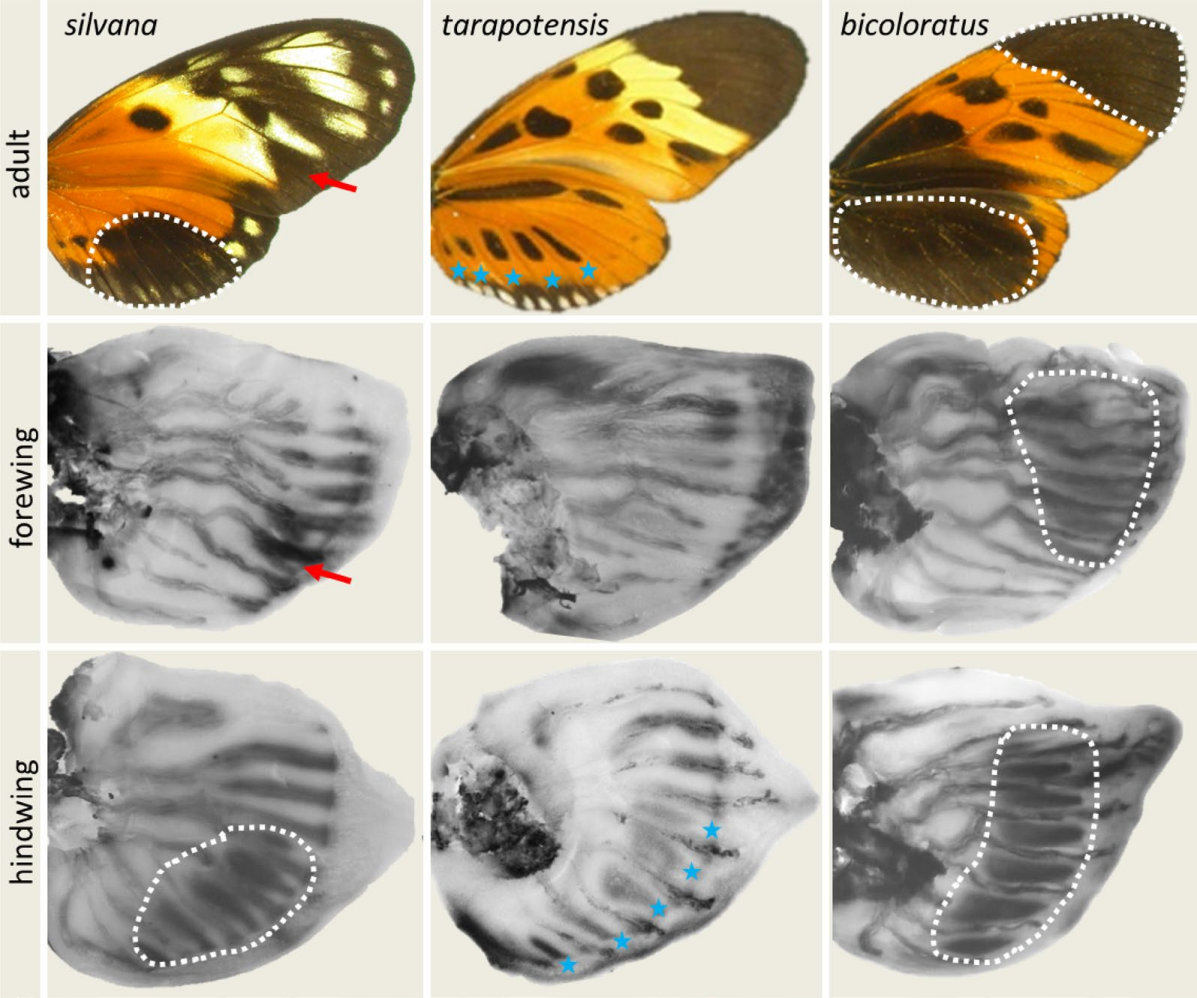
Expression patterns of *cortex* in larval wing discs of different *H. numata* morphs. Expression of *cortex* in late larval wing discs is associated with the amount of black pigment in the hindwings (top and bottom panels, stars and dashed lines indicate corresponding areas of the wings), the “comma mark” on *silvana* forewing (red arrows), and the black forewing tip in *bicoloratus*

To establish the relationships between expression and adult wing patterns, we performed in situ hybridization (ISH) with probes against 28 candidate genes (see Additional file 1: Table S1) from the *P* supergene. As the prepupal and day-1 pupal wings are too fragile to be used in ISH, we performed the latter on the wing discs from the last instar larvae. For all but one candidate, ISH resulted either in the ubiquitous expression or in the absence of any detectable signal (see Additional file 1: Figure S2 for examples). However, in line with the previous study [20], clear differences in the spatial patterns of expression were observed for the *cortex* gene (Fig. 2 and Additional file 1: Figure S3). In particular, the pattern of *cortex* signal in larval hindwings reflects the amount of black pigment in the adults. Moreover, *cortex* is expressed in the black forewing tip in *bicoloratus* (but not in other morphs), and in the so-called “comma mark” on the forewing of *silvana*. These results are consistent with the findings of the differential expression analyses, e.g. high expression levels of *cortex* in *bicoloratus* and low in *tarapotensis* at the prepupa stage.

## Discussion

In addition to developing the first transcriptome resource for *H. numata*, we performed differential expression analyses at two developmental stages to identify genes involved in variation of wing colour patterns in this species. A large number of genes throughout the transcriptome were differentially expressed among *H. numata* morphs: 1048 (out of 53,719) at prepupa and 3488 at day-1 pupa stage, providing a long list of putative candidates. However, because variation in *H. numata* colour pattern is known to be controlled by a single Mendelian locus located in the *P* region [26], these transcriptional differences could be explained by (a) downstream responses to signals coming from the supergene and co-segregating parts of the genome and/or (b) physiological response to fluctuating field conditions in Peru. This highlights the need for mapping the loci of interest prior to differential expression analyses in order to limit the number of candidate genes identified by RNA sequencing, and target the causative region.

By specifically focusing on the *P* locus, previously shown to control colour pattern variation in *H. numata* [7], our experiments, even though they were limited by a number of morphs, replicates, and developmental time points, confirm the role of *cortex* in the variation of some black pattern elements on the wings across *H. numata* morphs. Colour pattern phenotypes caused by *cortex* expression in *H. melpomene* and *H. numata* strikingly differ, suggesting that the emergence of novel patterns is caused by heterotopic expression, i.e. changes in the spatial regulation of *cortex*. Similar to *optix* which causes variation in red pattern elements [45], the evolution of black colour patterns within and among *Heliconius* species may be mainly driven by evolution of regulatory regions, changing the localisation of gene expression throughout the wing which is, in turn, determined by upstream positional signals. As in *H. melpomene*, differential expression was not confined to *cortex* exons [20], as 22 transcripts up- or downregulated at the prepupa stage map to its first intron and could indicate unidentified splice variants or non-coding RNAs.

We also found that the majority of genes in the *P* interval were expressed in the developing wing discs during the critical stages of colour pattern formation, and nine of them were expressed differentially among morphs. In addition, expression levels of several contigs corresponding to intronic and intergenic regions correlate with the presence/absence of the chromosomal inversions within the *P* interval. Large genomic rearrangements are frequently associated with gene silencing or ectopic expression patterns (e.g. [46, 47]). Additional studies of gene expression and function will investigate whether these changes indeed play a role in colour pattern variation in *H. numata*.

Most of the genes within the *P* interval are expressed in (pre)pupal wing discs, and quantitative as well as qualitative variation in expression of multiple genes has been detected between *P* genotypes, suggesting that inversions are associated with drastic changes in expression throughout the *P* region. Furthermore, variation in *cortex* expression revealed by ISH is restricted to certain parts of the wing and therefore does not fully recapitulate the variation in adult wing pattern, suggesting that other genes located in the *P* interval may play a role in colour pattern variations, independently of variation in black elements observed across *H. numata* morphs. The technical limit of ISH focusing on the restricted developmental windows may prevent us to detect other genes involved in the developmental switch between mimetic patterns in *H. numata*. Identifying the functional role of *cortex* using CRISPR/Cas9 knockout, for instance, would help understanding its implication in the development of different colour patterns in *H. numata* and its putative interactions with other genes.

Nevertheless, our current results are overall consistent with the hypothesis that the *P* locus acts as a classical supergene, whereby several linked genes are involved in the developmental switch between the different mimetic patterns. It should be noted that the second inversion has been recently shown to be followed by a third rearrangement, whose limits are not fully determined yet (unp. data), so that genes located further away from the region studied here could also be involved in the developmental switch.

Even though our ISH experiments performed at late larval stage cannot fully demonstrate the supergene hypothesis, our study opens up new perspectives on the architecture of the *P* locus in *H. numata*. Because of the technical constraints of ISH on wing disc, we studied only a limited time window of wing pattern formation. However, variation in colour patterns could be triggered by heterochrony in gene expression, or differential expression at stages not targeted here. Further studies on extended developmental time series are required to uncover full genetic control of such striking variation and to investigate the genetic architecture of the *P* supergene.

## Conclusions

Here, we developed the first transcriptome resource for *H. numata* and performed differential expression analyses during wing development to identify genes involved in forming the supergene and contributing to the expression of differentiated phenotypes. Our analyses confirm the role of *cortex* in the formation of black wing pattern elements. Our results are consistent with the hypothesis that other genes in the *P* region may play a role in colour variation in *H. numata*. Functional studies on the gene *cortex* and exploration of longer developmental time series will now be required to conclude whether the supergene *P* is a classical supergene, or whether *cortex* alone fully controls the developmental switches involved in colour pattern polymorphism in *H. numata*.

## Additional files

**Additional file 1: Table S1.** Information on probes used for ISH in *H. numata* larval wing discs. **Figure S1.** Principal component analysis (PCA) plot of read counts matrix from RNA-seq data. **Table S2.** Gene set enrichment analysis comparing the rank of differential expression in transcripts mapped to the supergene P to the rest of the transcriptome using 1,000 transcript permutations. **Table S3.** Analysis of splicing sites in the first intron of *cortex*. **Figure S2.** Examples of expression patterns observed in larval wing discs of *H. numata.* A. Ubiquitous expression, B. Expression in the trachea, C. No detectable signal. **Figure S3.** Expression patterns of *cortex* in larval wing discs of *H. numata* (all samples).

## Data Availability

The datasets generated and analysed during the current study are available in the NCBI SRA and TSA repositories (PRJNA555830).
