# Supplementary material for: Unravelling the genes forming the wing pattern supergene in the polymorphic butterfly Heliconius numata
Source: EvoDevo. 2019 Aug 8;10:16. doi: 10.1186/s13227-019-0129-2 (PMC6686539; doi:10.1186/s13227-019-0129-2)
Supplement: Supplementary file 1 — Additional file 1: Table S1. Information on probes used for ISH in H. numata larval wing discs. Figure S1. Principal component analysis (PCA) plot of read counts matrix from RNA-seq data. Table S2. Gene set enrichment analysis comparing the rank of differential expression in transcripts mapped to the supergene P to the rest of the transcriptome using 1,000 transcript permutations. Table S3. Analysis of splicing sites in the first intron of cortex. Figure S2. Examples of expression patterns observed in larval wing discs of H. numata. A. Ubiquitous expression, B. Expression in the trachea, C. No detectable signal. Figure S3. Expression patterns of cortex in larval wing discs of H. numata (all samples). [file 13227_2019_129_MOESM1_ESM.docx]

**Supplementary Figures and Tables**

**Supplementary Table S1 – Information on probes used for ISH in *H. numata* larval wing discs**

| **Contig ID** | **Hmel orthologue** | **Putative gene name** | **Primers (F – forward, R – reverse)** | **Expression pattern** |
| --- | --- | --- | --- | --- |
| comp41551_c0_seq1 | 000020 | glutaminyl-peptide cyclotransferase | F: CACGAGGCACAAGAACTGAC  R: GGCGGAAAGTTGACCCAATT | trachea |
| comp46319_c0_seq1 | 000022 | enoyl-CoA hydratase | F: GCCTCGCCGTTCTTTCAAAT  R: TGACTGCTTGTTTCGCCATC | ubiquitous |
| comp35224_c0_seq1 | 000024 | Sur-8/LRR | F:GGAAATGGAGGAGAATGAAAACA R: AAGTTCTGATGTCCCCAAACA | no signal |
| comp452182_c0_seq1 | 002024 | Hmel002024 | F: GGTTCGCCCAAATCCTACAC  R: GGGTCGAGATGCCAAGTTTG | trachea |
| comp45148_c0_seq1 | 000025 | cortex | F: ATCCATTTTGACATCGGGCG  R: CATGTTGTCGTTGCTCCAGT | see Figure 2 |
| comp47446_c0_seq1 | 000026 | poly(A)-specific ribonuclease (parn) | F: TGGGAAGAGTTTGAGGAAGCT  R: CGCTGTCGTTTCATTGAGGA | ubiquitous |
| comp43223_c0_seq1 | 000027 | Hmel000027 | F: CAAGACTTCGTTGTGCCCAA  R: CAGAACACACTCGAACAGGC | ubiquitous |
| comp29037_c0_seq1 | 000028 | ARP-like | F: AAAGAAGGCGAGTGTGAAGT  R: TCCTCTTGATGAAGTCGGTCT | ubiquitous |
| comp28890_c0_seq1 | 000029 | ATP synthase subunit f, mitochondrial | F: GCATTCGGTGATTATCCCAAAGA  R: TGTCATTGATCCAACCAGCAG | ubiquitous |
| comp34317_c0_seq1 | 000030 | proteasome 26S non ATPase subunit 4 | F: ACTTCATCGTGTCCAACCCA  R: CCATAGCTTCCCTCCCAAGA | ubiquitous |
| comp46944_c0_seq1 | 000032 | zinc phosphodiesterase | F: ACCGCCACATGTGTTTAACC  R: TTCGGTCCGTAGAAGCGTAC | trachea |
| comp46606_c0_seq1 | 000033 | serine/threonine-protein kinase | F: GCCATAATACACGCCCCATC  R: TCATCATCACAAGGCTCGGT | ubiquitous |
| comp36207_c0_seq1 | 000036 | WAS protein family homologue 1 | F: AACACGGACAAGACAGCAAA  R: GTTCTGACAATTCGGGCAAT | ubiquitous |
| comp46812_c0_seq1 | 000037 | tyrosine phosphatase (Domeless) | F: GTCGAAAGTCCTCCAGCAGA  R: TGTAGCGTGGTCAAATGCTC | trachea |
| comp45489_c0_seq1 | 000038 | lethal (2) k05819 CG3054 | F: AGTGAGAAAATGGGCTTGGA  R: GACTTGGTACGGTGGCATCT | no signal |
| comp44264_c0_seq1 | 000039 | mitogen-activated protein kinase (MAPKK) | F: CCACAGGCCCAGTAATACCA  R: AGTTAGGCCGCTGCTTGTAA | ubiquitous |
| comp29763_c0_seq1 | 000040 | DNA excision repair protein ERCC-6 | F: CCAAATCTGAGGGTTGCTGT  R: TCGGGATGGTTGCATATTTT | trachea |
| comp43930_c0_seq1 | 000041 | penguin | F: TGCCCACCATTCTGTAAAGC  R: ACCCCAATGATCAGCAGCTA | ubiquitous |
| comp39814_c0_seq1 | 000042 | thymidylate kinase | F: GCAAGTATCAGCCGAATACATGA  R: ACACCGTCTACAACTGATAGC | ubiquitous |
| comp51539_c0_seq1 | 000043 | caspase-activated DNase | F: AAATTGCCGTCTTTTTGTGGC  R: TCGTTCCTCGCGACTTCTAT | ubiquitous |
| comp43148_c0_seq1 | 000044 | ribosome biogenesis regulatory protein | F: GCGATTGATGAAGCGATCGT  R: TGCTGTGGATAGCTGAGTGG | ubiquitous |
| comp39269_c0_seq1 | 000045 | INO80 complex subunit C | F: GCAGTGGTAAGCATGAGTGG  R: GTTGCCCCTCGTAATTGAAGA | ubiquitous |
| comp42391_c0_seq1 | 032684 | uncharacterized WD repeat-containing protein C2E1P5.05 | F: ATGTGGCACCAAAGGAAAGTG  R: TCTCGACCACCTGATGTGATG | ubiquitous |
| comp42658_c0_seq1 | 000047 | Sr protein | F: GGTCTCACTCGGACTCGAAG  R: TTGGTCGTAAAATGCCCAATT | ubiquitous |
| comp10602_c0_seq1 | 000048 | no data | F: CACAAAGAAGGCGAATTGCTC  R: GGATTTGTTTGGGACGAGTG | ubiquitous |
| comp166797_c0_seq1 | 000049 | no data | F: AACTACCAATGGCACGAAGA  R: CCAGGTGTTGTGCAAAGTGTG | no signal |
| comp41872_c0_seq1 | 000050 | shuttle craft | F: GGCCCAAAAGTTCTCCATCAA  R: GAAGCTGACACCACAGACGA | ubiquitous |
| comp46938_c0_seq1 | 000053 | lethal (2) giant larva | F: GATTGGCTGTTAAGCGGGTAC  R: TTCCACAGCACGCTCTATTGG | ubiquitous |

**Supplementary Figure 1 - Principal component analysis (PCA) plot of read counts matrix from RNAseq data**


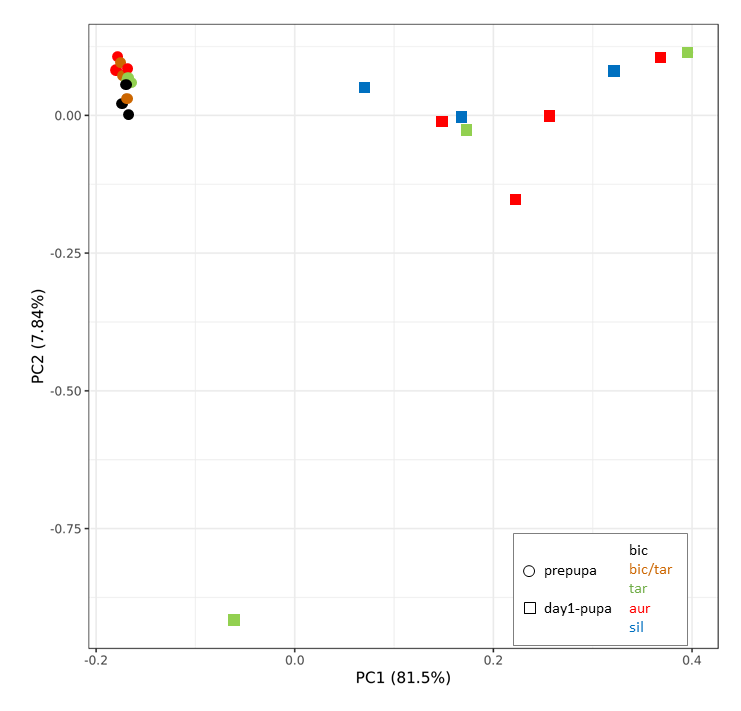


**Supplementary Table S2 : Gene-Set Enrichment Analysis comparing the rank of differential expression in transcripts mapped to the supergene P to the rest of the transcriptome using 1,000 transcript permutations**.

Analyses were performed by comparing transcript expression between two genotypes at the supergene within a developmental stage (Note that the number of transcripts mapped to the supergene differs between stages). **ES**: enrichment score, **NES**: enrichment score normalized by the number of transcripts mapped to the supergene at a given developmental stage, **P-value**: nominal P-value obtained using 1,000 permutations of transcripts throughout the rest of the transcriptome, **Leading edge tag**: estimations of the percentage of transcripts within the supergene contributing to the enrichment score. These analyses were carried out using the GSEA software (<http://software.broadinstitute.org/gsea/>) applied to edgeR differential expression analysis outputs.

| **Stage** | **Comparisons** | **# transcripts** | **ES** | **NES** | **P-value** | **Leading edge tags** |
| --- | --- | --- | --- | --- | --- | --- |
| prepupae | aur vs. bic | 191 | -0.289 | -1.285 | 0.035 | 24% |
| prepupae | aur vs. tar | 191 | 0.378 | 1.657 | <0.001 | 26% |
| prepupae | bic vs. tar | 191 | 0.418 | 1.848 | <0.001 | 28% |
| prepupae | bic/tar vs. aur | 191 | -0.423 | -1.756 | <0.001 | 32% |
| prepupe | bic/tar vs. bic | 191 | -0.193 | -0.905 | 0.748 | 16% |
| prepupe | bic/tar vs. tar | 191 | -0.523 | -2.293 | <0.001 | 35% |
| 24h | aur vs. sil | 177 | 0.547 | 2.200 | <0.001 | 38% |
| 24h | tar vs. aur | 177 | -0.274 | -1.337 | 0.032 | 31% |
| 24h | tar vs. sil | 177 | 0.524 | 2.453 | <0.001 | 37% |

**Supplementary Table S3 - Analysis of splicing sites in the first intron of *cortex***

| **Transcript** | **Length (bp)** | **Best hit to H.numata scaffold (Lepbase v4)** | **5' flanking sequence (20 bp)** | **3' flanking sequence (20 bp)** |
| --- | --- | --- | --- | --- |
| comp388498_c0_seq1 | 336 | heliconius_numata_helico3_core_32_85_1__scaffold__EI-a-scaffold-12004 | CGTATTGTAATACAATATTA | GACGTTCATGCAATGTATGA |
| comp407410_c0_seq1 | 213 | heliconius_numata_helico3_core_32_85_1__scaffold__EI-a-scaffold-9216 | ATTGTAATGAAATGAATACG | GCCCCACTTCCTGACGCCCA |
| comp398064_c0_seq1 | 363 | heliconius_numata_helico3_core_32_85_1__scaffold__EI-a-scaffold-9216 | CCAAGTCGTTATAATTTTAC | NNNNNNNNNNNNNNNNN |
| comp601770_c0_seq1 | 228 | heliconius_numata_helico3_core_32_85_1__scaffold__EI-a-scaffold-12846 | TATATTTTACATAATATTTT | GGACGAATAACTCAATATCA |
| comp415437_c0_seq1 | 436 | heliconius_numata_helico3_core_32_85_1__scaffold__EI-a-scaffold-12846 | TATTAATAAATAATAGTACA | CCGGAGAATTCTGGAGGTAC |
| comp423466_c0_seq1 | 222 | heliconius_numata_helico3_core_32_85_1__scaffold__EI-a-scaffold-12846 | ATTTCAAATATTGAGTAAAA | AAGTTTACCCCGTTCCGTCA |
| comp407210_c0_seq1 | 340 | heliconius_numata_helico3_core_32_85_1__scaffold__EI-a-scaffold-12846 | ACAGCAATAAGTAAATACAA | AAATCCAAGACAATAGCTTT |
| comp467980_c0_seq1 | 279 | heliconius_numata_helico3_core_32_85_1__scaffold__EI-a-scaffold-12846 | AAATATCAATATGATCCAAA | GGTGCGCTATCTTGCTATAG |
| comp472259_c0_seq1 | 209 | heliconius_numata_helico3_core_32_85_1__scaffold__EI-a-scaffold-12846 | TGAAATTAAATGAATAATTC | GGAACTTAGTGTTCAGACAT |
| comp700774_c0_seq1 | 227 | heliconius_numata_helico3_core_32_85_1__scaffold__EI-a-scaffold-12846 | GCAGTAAATTTCAGTTTCTA | CAAACTTAGGTAAGGCTATG |
| comp321273_c0_seq1 | 390 | heliconius_numata_helico3_core_32_85_1__scaffold__EI-a-scaffold-12846 | AATCAAGATTTTATCATAGC | AAGTGAGGAAACCTAAATAG |
| comp62384_c0_seq1 | 396 | heliconius_numata_helico3_core_32_85_1__scaffold__EI-a-scaffold-12846 | AAATGAAATGAAAGACATTT | CTGACTGAGGTAAGACGCAC |
| comp31128_c1_seq2 | 643 | heliconius_numata_helico3_core_32_85_1__scaffold__EI-a-scaffold-12846 | CATGGTTTATTATAATTCAC | CGTATAAAAGAGCCGGTCAA |
| comp83470_c0_seq1 | 841 | heliconius_numata_helico3_core_32_85_1__scaffold__EI-a-scaffold-12846 | AAAAAAAACATTATTTATCA | CCGTGTGTTTTTATATAACT |
| comp90384_c0_seq1 | 240 | heliconius_numata_helico3_core_32_85_1__scaffold__EI-a-scaffold-12846 | AAAGTGATTTGAGATTTCTG | ACCGCTCCCGCGCCTCTTGT |
| comp382243_c0_seq1 | 295 | heliconius_numata_helico3_core_32_85_1__scaffold__EI-a-scaffold-12846 | ATATTCGAAGAGGCCAGTCC | TGTTTATATCATCAGGTTAA |
| comp144920_c0_seq1 | 281 | heliconius_numata_helico3_core_32_85_1__scaffold__EI-a-scaffold-12846 | TTTTTTATGAAAGTTTTAAA | CTGTATTTTTTTACTACCCG |
| comp615571_c0_seq1 | 265 | heliconius_numata_helico3_core_32_85_1__scaffold__EI-a-scaffold-12846 | CATAATACAAAGTGATCCTA | ACTGCCACTGGAGTTGGGCT |
| comp386445_c0_seq1 | 491 | heliconius_numata_helico3_core_32_85_1__scaffold__EI-a-scaffold-12846 | AAAAAATGTATTGATATGGC | TATATACGGAAACACCATCT |
| comp527091_c0_seq1 | 245 | heliconius_numata_helico3_core_32_85_1__scaffold__EI-a-scaffold-12846 | TATAAATCACGAAAGATGAG | AAAAAAAATTCTATACTAAA |
| comp572350_c0_seq1 | 368 | heliconius_numata_helico3_core_32_85_1__scaffold__EI-a-scaffold-12846 | CAACATTTAAAAAAAATTCT | ATTTGACCTCATTTAGTAAT |
| comp331233_c0_seq1 | 230 | heliconius_numata_helico3_core_32_85_1__scaffold__EI-a-scaffold-12846 | ACAAATTTGTAGTTACAGTT | TTTTAAAGAGACAAACATCG |

**Supplementary Figure S2 – Examples of expression patterns observed in larval wing discs of *H. numata***

A. Ubiquitous expression, B. Expression in the trachea, C. No detectable signal.

**
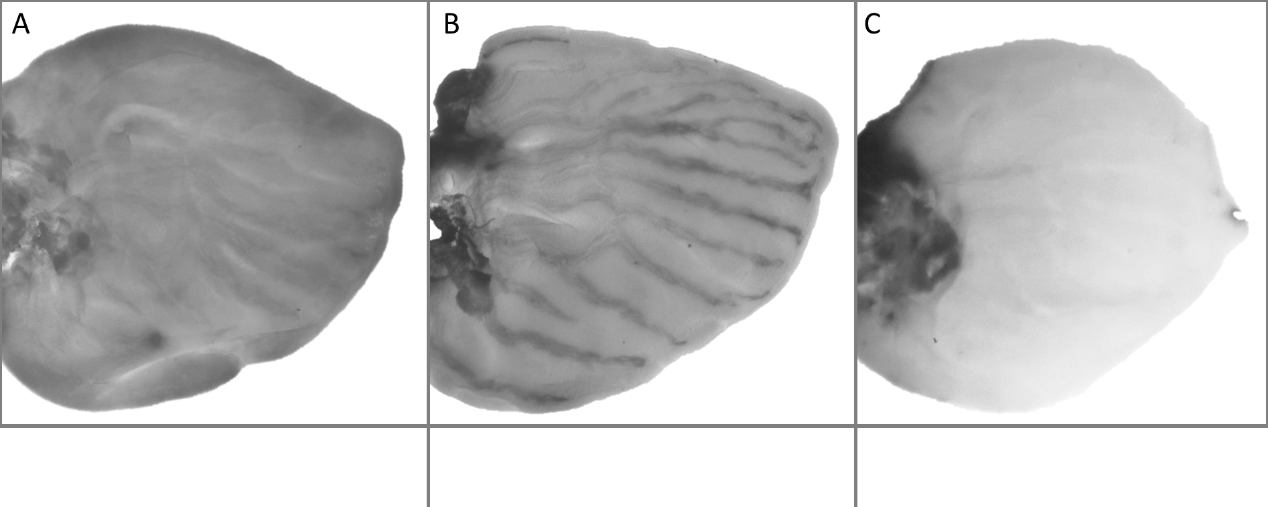
**

## **Supplementary Figure S3 - Expression patterns of *cortex* in larval wing discs of *H. numata* (all samples)**

**
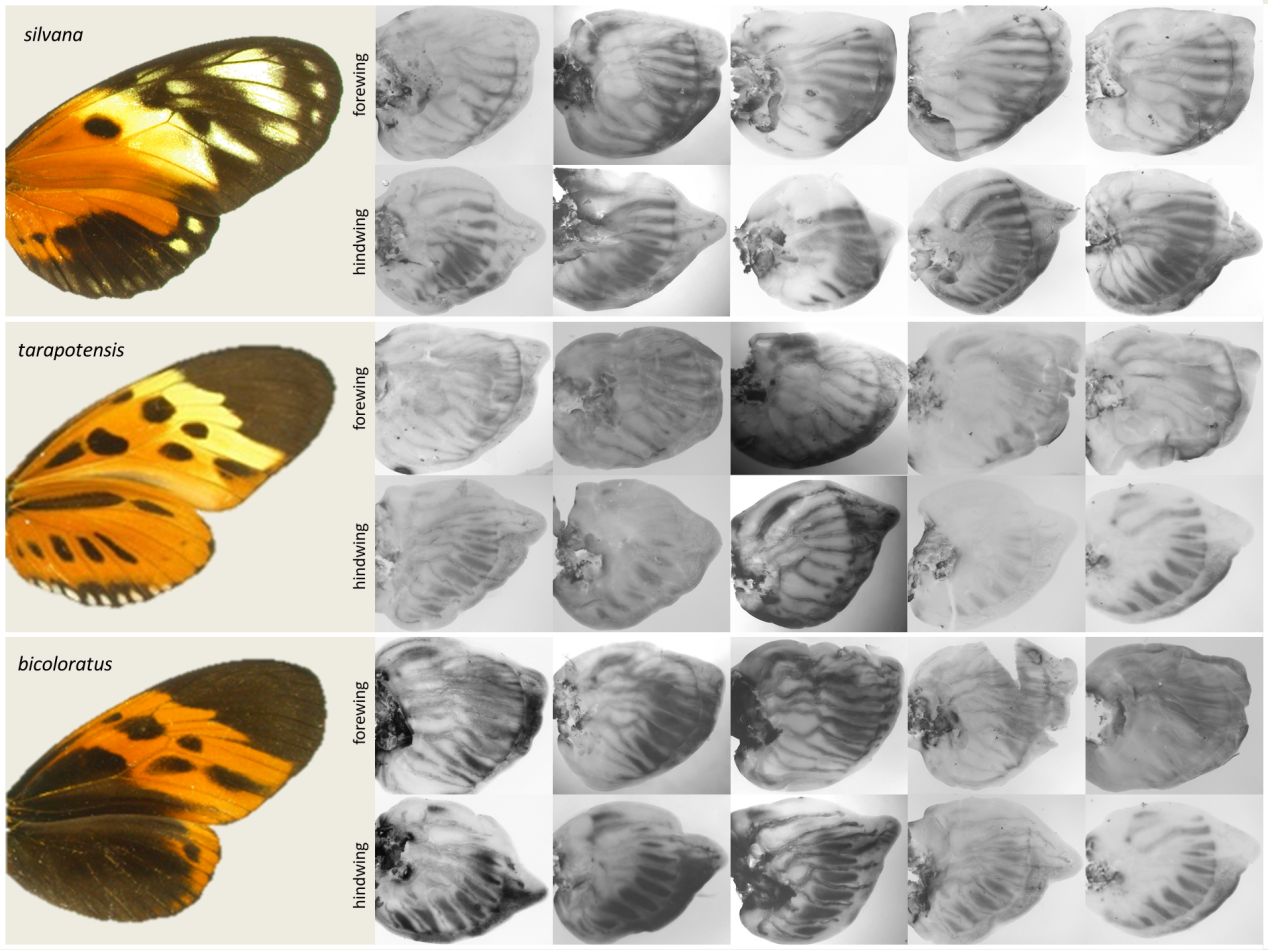
**
